# Metal Binding of Alzheimer’s Amyloid-β and Its Effect on Peptide Self-Assembly

**DOI:** 10.1021/acs.accounts.3c00370

**Published:** 2023-09-21

**Authors:** Axel Abelein

**Affiliations:** Department of Biosciences and Nutrition, Karolinska Institutet, 141 52 Huddinge, Sweden

## Abstract

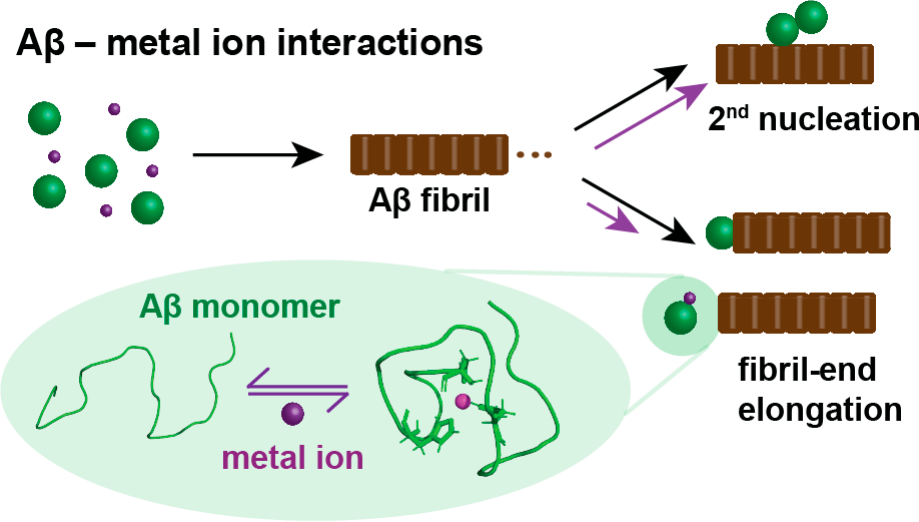

Metal ions have been identified as key factors modulating the aggregation of amyloid-β peptide (Aβ) implicated in Alzheimer’s disease (AD). The presence of elevated levels of metal ions in the amyloid plaques in AD patients supports the notion that the dysfunction of metal homeostasis is connected to the development of AD pathology. Here, recent findings from high- and low-resolution biophysical techniques are put into perspective, providing detailed insights into the molecular structures and dynamics of metal-bound Aβ complexes and the effect of metal ions on the Aβ aggregation process. In particular, the development of theoretical kinetic models deducing different microscopic nucleation events from the macroscopic aggregation behavior has enabled deciphering of the effect of metal ions on specific nucleation processes. In addition to these macroscopic measurements of bulk aggregation to quantify microscopic rates, recent NMR studies have revealed details about the structures and dynamics of metal-Aβ complexes, thereby linking structural events to bulk aggregation. Interestingly, transition-metal ions, such as copper, zinc, and silver ions, form a compact complex with the N-terminal part of monomeric Aβ, respectively, where the metal-bound “folded” state is in dynamic equilibrium with an “unfolded” state. The rates and thermodynamic features of these exchange dynamics have been determined by using NMR relaxation dispersion experiments. Additionally, the application of specifically tailored paramagnetic NMR experiments on the Cu(II)-Aβ complex has been fruitful in obtaining structural constraints within the blind sphere of conventional NMR experiments. This enables the determination of molecular structures of the “folded” Cu(II)-coordinated N-terminal region of Aβ. Furthermore, the discussed transition-metal ions modulate Aβ self-assembly in a concentration-dependent manner, where low metal ion concentrations inhibit Aβ fibril formation, while at high metal ion concentrations other processes occur, resulting in amorphous aggregate formation. Remarkably, the metal-Aβ interaction predominately reduces one specific nucleation step, the fibril-end elongation, whereas primary and surface-catalyzed secondary nucleation mechanisms are less affected. Specific inhibition of fibril-end elongation theoretically predicts an enhanced generation of Aβ oligomers, which is an interesting contribution to understanding metal-Aβ-associated neurotoxic effects. Taken together, the metal binding process creates a metal-bound Aβ complex, which is seemingly inert to aggregation. This process hence efficiently reduces the aggregation-prone peptide pool, which on the macroscopic level is reflected as slower aggregation kinetics. Thus, the specific binding of metals to the Aβ monomer can be linked to the macroscopic inhibitory effect on Aβ bulk aggregation, providing a molecular understanding of the Aβ aggregation mechanism in the presence of metal ions, where the metal ion can be seen as a minimalist agent against Aβ self-assembly. These insights can help to target Aβ aggregation *in vivo*, where metal ions are key factors modulating the Aβ self-assembly and Aβ-associated neurotoxicity.

## Key References

- AbeleinA.; Ciofi-BaffoniS.; MörmanC.; KumarR.; GiachettiA.; PiccioliM.; BiverstålH.Molecular Structure of Cu(II)-Bound Amyloid-β Monomer Implicated in Inhibition of Peptide Self-Assembly in Alzheimer’s Disease. JACS Au2022, 2, 2571–258410.1021/jacsau.2c00438.36465548PMC9709942(1)*This is the first study providing molecular structures of the Cu(II)-Aβ complex using specifically tailored paramagnetic NMR experiments and molecular dynamics simulations. Furthermore, by applying a detailed kinetics analysis, a specific effect of Cu(II) on the Aβ aggregation mechanism was shown.*
- AbeleinA.; GräslundA.; DanielssonJ.Zinc as chaperone-mimicking agent for retardation of amyloid β peptide fibril formation. Proc. Natl. Acad. Sci. U. S. A.2015, 112, 5407–54122582572310.1073/pnas.1421961112PMC4418866.^2^*This study pioneered the application of theoretical kinetic models to describe the modulation of Aβ aggregation kinetics by metal ions, here applied for Zn(II). Furthermore, detailed insights into the dynamics and thermodynamics of metal ion binding were reported.*
- WallinC.; JarvetJ.; BiverstålH.; WärmländerS.; DanielssonJ.; GräslundA.; AbeleinA.Metal ion coordination delays amyloid-beta peptide self-assembly by forming an aggregation-inert complex. J. Biol. Chem.2020, 295, 7224–723410.1074/jbc.RA120.01273832241918PMC7247290.^3^*This is a detailed investigation of the binding of monovalent Ag(I) ions to Aβ, compared to divalent Zn(II) ions, which showed the molecular properties of metal binding and its effect on the aggregation mechanism, revealing a specific effect on fibril-end elongation.*

## 1 Introduction

The misfolding of proteins and peptides is suspected to cause several devastating neurodegenerative disorders, among them the most prevalent one Alzheimer’s disease (AD).^4^ The mainly 40- or 42-residue-long amyloid-β peptide (Aβ) is processed from the amyloid-β precursor protein by enzymatic cleavage.^5^ Besides the most frequently occurring Aβ40 and Aβ42 isoforms, also other N-terminally truncated forms, such as Aβ(3-42) and Aβ(4-40) have recently been identified in human cerebrospinal fluid (CSF).^6^ The predominantly unstructured Aβ40 and Aβ42 monomers can subsequently aggregate into mature, β-structured amyloid fibrils, which are the main components of amyloid plaques found in AD patients’ brains.^5^ The hydrophobic middle and C-terminal parts of Aβ build up the core of the fibril structure (Table 1).^7^ Notably, increasing lines of evidence indicate that the most toxic species is presumably not the mature amyloid fibril as such but smaller oligomeric species that occur prior to fibril formation.^8^ Furthermore, new treatment approaches targeting specific steps in the Aβ aggregation process have led to positive treatment results.^9,10^

**Table 1:**
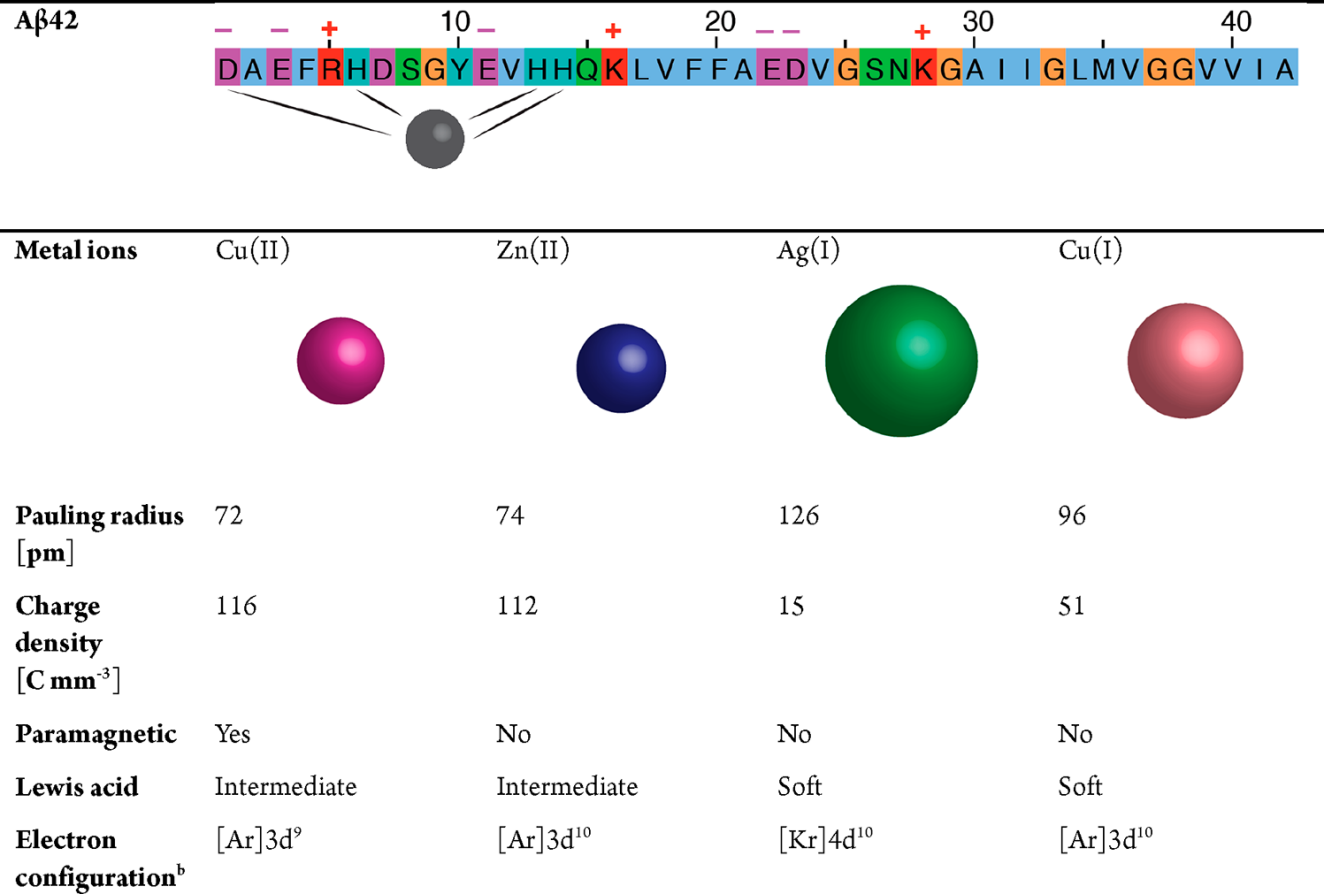
Fundamental Properties of Aβ and Selected Transition-Metal Ions^a^

a (Top) Aβ42 sequence color-coded for negative (magenta), positive (red), hydrophobic (blue), polar (green), aromatic (cyan), and glycine (orange) residues, where major metal binding ligands are marked. (Bottom) Properties of Cu(II), Zn(II), Ag(I), and Cu(I) ions (adapted from ref (3) with references therein). The sizes of the depicted metal ions reflect their Pauling radii.

b d^9^ exhibits Jahn−Teller effect to stabilize ligand binding.

Metal ions are an essential part of the cellular system and are crucial for the correct function of diverse metalloproteins.^11^ The metal homeostasis involves numerous proteins, such as metallothionein, and processes that transport and buffer metal ions and hence determine their bioavailability.^12−14^ Remarkably, metal ions also appear as key players modulating protein self-assembly and associated toxicity, including for Aβ, which has been the subject of several comprehensive review articles.^13,15−21^ Elevated levels of copper and zinc ions were found in amyloid plaques of AD patients, suggesting a link to AD development.^22−24^ How metal ion concentrations are modulated in the brain has been difficult to assess, yet a dysfunction of metal homeostasis is apparently connected to AD progression, as previously reviewed in refs (25) and (26) and references therein. Furthermore, copper complexes with different Aβ isoforms have been reported in CSF,^6^ and specific copper chelators have been shown to modulate AD brain damage, as reviewed in ref (27). Hence, there is strong support that physiological metal ions, such as copper and zinc, are implicated in AD.^12^ These metal ions bind monomeric Aβ in the N-terminal part (Table 1) and modulate the Aβ aggregation pathway (*vide infra*). In the case of copper, the formation of neurotoxic reactive oxygen species (ROS) can be triggered, which contributes to Aβ-associated neurotoxic processes.^13,16^ In particular, in the synaptic cleft the concentrations of copper and zinc ions are unusually high, creating a possible hotspot for metal–Aβ interactions.^13,16^ Furthermore, other metal ions, such as silver ions, Ag(I), have mainly been studied as model metal ions, e.g., to investigate the effect of charge for monovalent vs divalent ions, but could also play a role through contamination.

The nucleation process of protein aggregation can be quantitatively described using an analytical solution of a set of differential equations,^28,29^ which has revealed detailed insights into the different microscopic rate constants governing Aβ aggregation.^30,31^ How the macroscopic effect of metal ions on modulating Aβ bulk aggregation can be deciphered into the modulation of microscopic nucleation events is the subject of this Account using the theoretical framework of kinetic equations. Furthermore, recent structural insights into the binding of metal ions, in particular, Cu(II), Zn(II), and Ag(I) ions (Table 1), including metal ion coordination and structural rearrangement and the link to its effect on Aβ aggregation, are discussed here.

## 2 Metal Ion Binding to Monomeric Aβ

**Figure 1:**
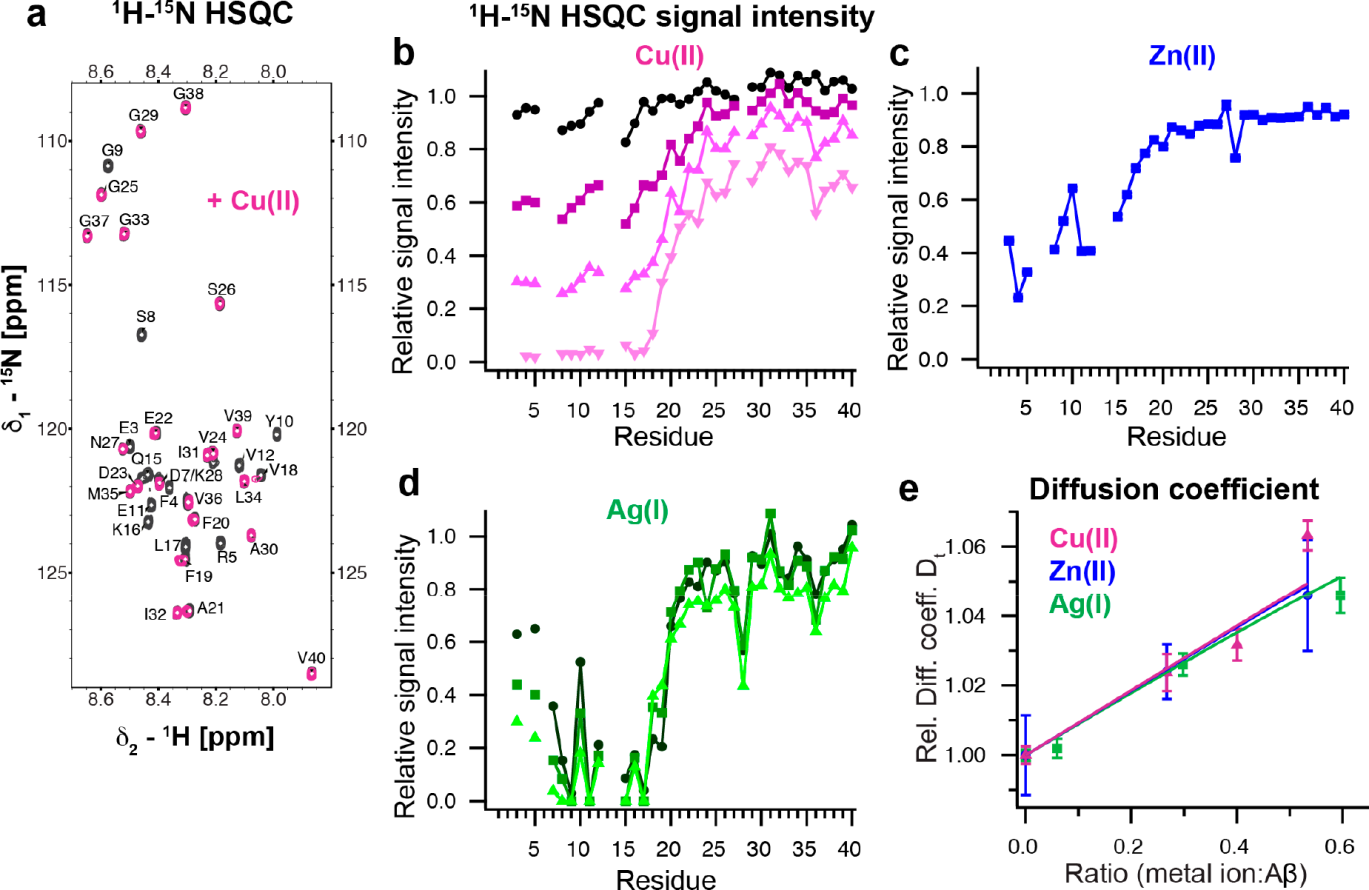
Metal ions specifically bind to the N-terminal part of Aβ and form a compact metal-Aβ complex. (a) ^1^H–^15^N HSQC spectrum of 75 μM Aβ40 in 10 mM HEPES, pH 7.2, with (violet) and without (black) 100 μM Cu(II) at 281 K.^1^ (b–d) Relative ^1^H–^15^N HSQC signal intensities of Aβ40 in the presence of different concentrations of Cu(II), Zn(II), and Ag(I). Metal ion concentrations were 40 (black-violet), 60 (dark violet), 75 (violet) and 100 μM (light violet) for Cu(II), 20 μM for Zn(II) (blue), and 20 (dark green), 40 (green) and 80 μM (light green) for Ag(I) for 75 to 80 μM Aβ40.^1−3^ (e) Relative translational diffusion coefficients of Cu(II), Zn(II), and Ag(I) with a global fit to a two-state model, where the diffusion coefficients for the “unfolded” and compact “folded” states are shared fitting parameters.^1^ Data were replotted from refs (1−3).

To understand the modulating effect of metal ions on Aβ self-assembly, several studies have investigated the coordination of different metal ions in monomeric Aβ. This Account focuses on recent findings about the molecular structure induced upon metal binding and the dynamic processes and puts them into context with previous results reviewed in refs (15), (16), (32), and (33). An overview of the elementary properties of the metal ions discussed here, Cu(II), Cu(I), Zn(II), and Ag(I), is provided in Table 1. Nuclear magnetic resonance (NMR) is a powerful technique to elucidate the metal ion binding to monomeric Aβ. Titrating these metal ions onto ^15^N-labeled Aβ40 causes signal attenuation of ^1^H–^15^N HSQC NMR cross-peak signals in the N-terminal part of Aβ40 (Figure 1a–d). In general, NMR signal broadening can originate from paramagnetic relaxation and/or line broadening due to chemical exchange dynamics on an NMR intermediate time scale. Among the metal ions discussed here, only Cu(II) ions exhibit paramagnetic properties (Table 1), suggesting the presence of two interchanging states. The detailed origin of the NMR signal decrease is discussed in the following sections. The formation of a more compact metal ion-bound state is further supported by a decreased hydrodynamic radius, which is reflected as an increased translational diffusion coefficient in NMR diffusion measurements (Figure 1e).^1−3^ Remarkably, the diffusion data could be fitted globally, indicating that all metal ions generate a similar N-terminal more compact fold in Aβ (Figure 1e).^1^ These results agree well with the first study reporting a decreased hydrodynamic radius using size-exclusion measurements for Cu(II) and Zn(II) and NMR diffusion experiments for Zn(II).^34^

### 2.1 Copper Ions

#### 2.1.1 Divalent Copper Ions Cu(II)

The coordination of Cu(II) by Aβ has been reported to adapt two different modes, depending on the pH, which has primarily been explored by electron paramagnetic resonance (EPR) studies using frozen samples, and the binding site in solution was confirmed by other techniques, such as solution NMR.^15,16,32,33^ At physiological and lower pH values (pH < 7.8) component I pre-exists, which includes the NH_2_ terminus, the backbone CO group of D1, the imidazole rings of H6, and H13 or H14. Coordination modes with H13 or H14 as the fourth ligand are both present, referred to as Ia and Ib, respectively.^33^ High pH (pH > 7.8) causes deprotonation of the D1-A2 amide bond, and component II has then been identified as the predominant coordination mode, which consists of the NH_2_ terminus, the deprotonated amide of A2, the CO group of A2, and one of the imidazole rings of H6, and H13 or H14.^33^

**Figure 2:**
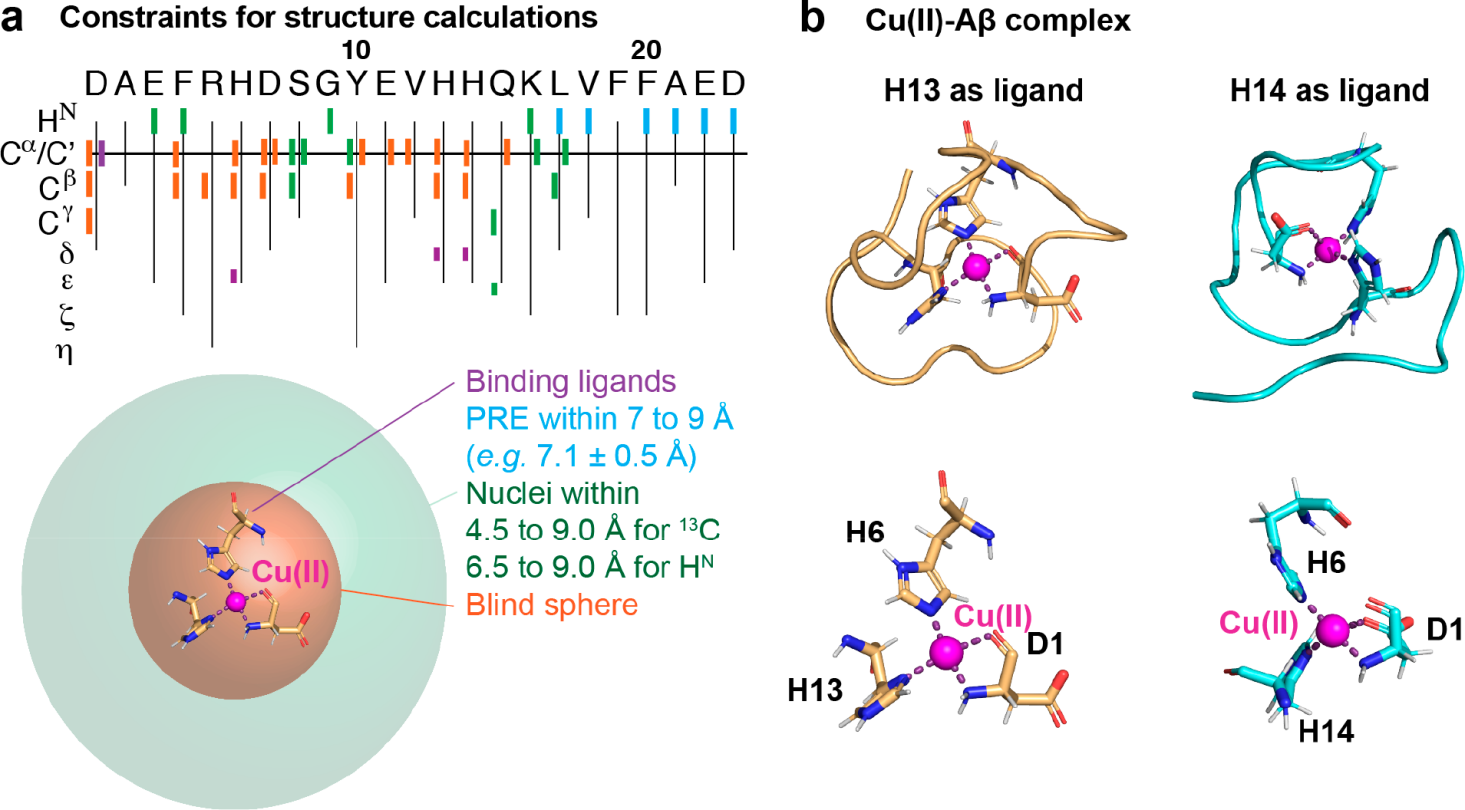
Molecular structures of the Cu(II)-Aβ complex using paramagnetic NMR experiments. (a) Structural constraints from different paramagnetic NMR experiments where binding ligands (violet) are within the blind sphere of paramagnetic NMR experiments (orange). Nuclei detected with paramagnetic NMR but not with diamagnetic NMR pulse sequences are located within the decreased blind sphere of paramagnetic compared to diamagnetic experiments (green). PRE measurements provide additional structural constraints (cyan). (b) Molecular structures of two different binding modes with H13 or H14 as the fourth binding ligand (available as PDB structure 8B9Q or 8B9R, respectively). The assigned binding ligands are the nitrogen of the NH_2_ terminus, the amide oxygen of D1, the N_ε_ of H6, and the N_δ_ of H13 or H14. Data were reproduced and the figure was adapted with permission from ref (1). Copyright 2022 the authors. American Chemical Society.

Recently, the application of paramagnetic NMR experiments revealed new insights into the molecular structures, confirming coordination mode I at physiological pH at 8 °C, where other alternative binding ligands, such as A2, E3, D7, Y10, and E11 could be excluded.^1^ The ^1^H–^15^N HSQC spectrum of a titration series of Cu(II) onto ^15^N-labeled Aβ40 exhibits a general signal attenuation of residues 1 to 21 in the N-terminal part (Figure 1a,b). Due to the paramagnetic nature of Cu(II), paramagnetic relaxation broadens the signals close to the paramagnetic center. Alongside the signal decrease, chemical shift changes of residues 18 to 21 were observed, indicating the presence of chemical exchange on the slow NMR time scale.^1^ In this study, a set of specifically tailored 2D and 3D paramagnetic NMR experiments and paramagnetic relaxation enhancement (PRE) experiments were applied to obtain signals from residues within the typical “blind sphere” of conventional NMR experiments (Figure 2a). A comparison of paramagnetic NMR with diamagnetic NMR experiments gives constraints for nuclei that are close enough to the paramagnetic center to be recorded by paramagnetic NMR but not by diamagnetic NMR measurements. Resonances that are not visible even in paramagnetic NMR experiments, such as direct binding ligands, can be constrained to the largely decreased blind sphere of paramagnetic NMR experiments (Figure 2a). The distance dependence of PREs can be directly translated to distance constraints. Structural calculations using these constraints, followed by structural refinement and molecular dynamics simulations, resulted in two structural models for Cu(II)-Aβ40 for the first 23 residues, with an average backbone RMSD to means of 1.92 and 2.13 Å for H13 and H14, respectively (Figure 2b and PDB IDs 8B9Q and 8B9R).^1^ Notably, the two binding ligands of the NH_2_ terminus and CO group of D1 theoretically allow two different chirality modes, where molecular dynamics simulations prefer one of them.^1^

#### 2.1.2 Monovalent Copper Ions Cu(I)

For monovalent Cu(I), which in contrast to Cu(II) does not exhibit paramagnetic properties (Table 1), a linear binding model was reported that includes the three histidines, where the Cu(I) ion is preferably coordinated by H13 and H14, in equilibrium with H6 and H13 or H6 and H14 coordination modes.^35,36^ Additionally, another binding mode where all three histidines act as ligands might be present.^35,36^ This model was obtained on Aβ(1–16) using ^1^H-detected NMR and X-ray absorption spectroscopy. Due to similar properties of Cu(I) and Ag(I), parts of the findings might be transferable, and indeed a similar coordination mode for Ag(I) as for Cu(I) was reported,^36^ indicating that Ag(I) has the potential to probe Cu(I)–Aβ interactions.

### 2.2 Zinc Ions Zn(II)

**Figure 3:**
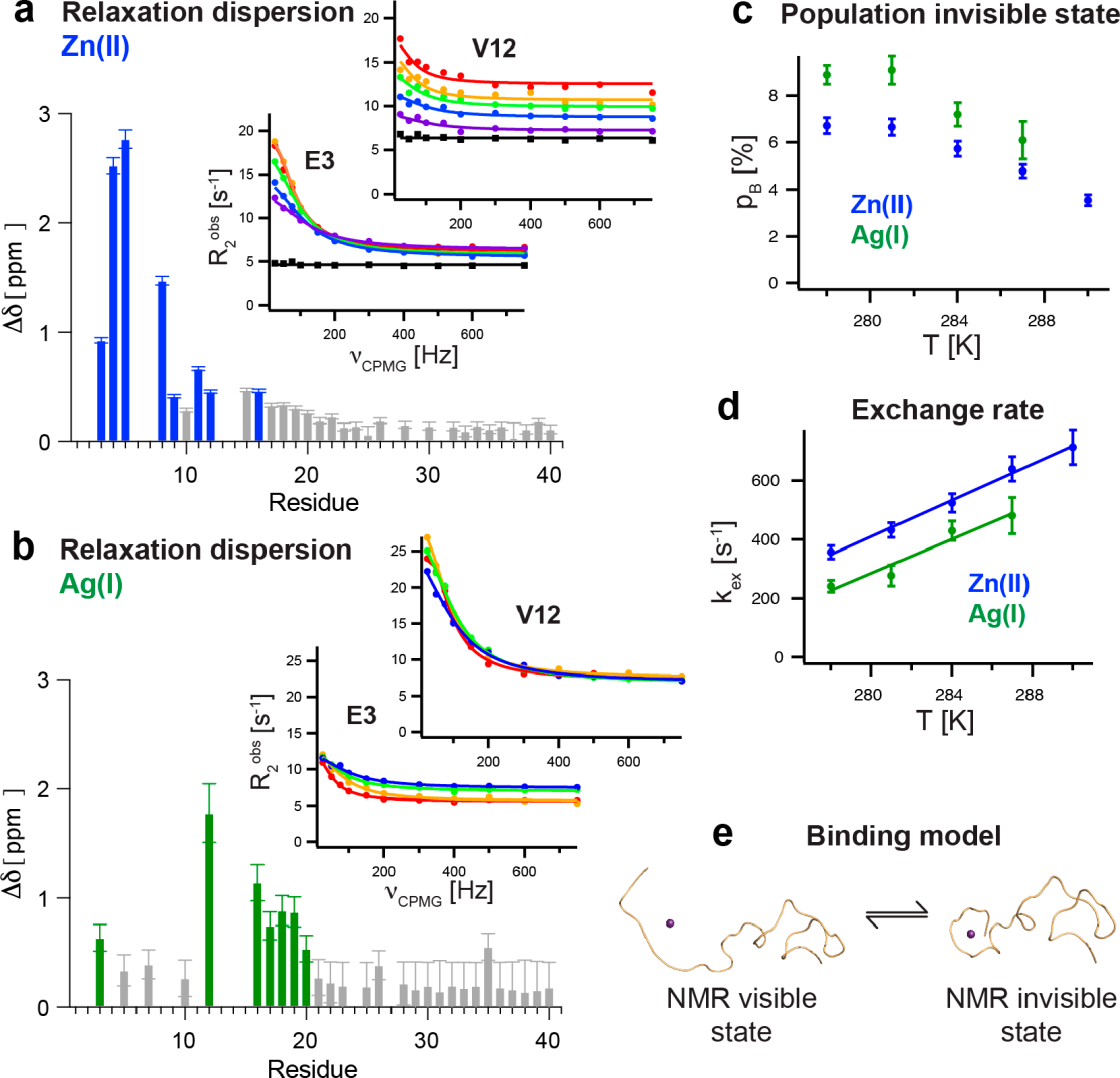
Exchange dynamics of Zn(II)- and Ag(I)-Aβ complexes characterized by NMR relaxation dispersion experiments. (a, b) Distinct residues exhibit significantly high amplitude ^15^N CPMG relaxation dispersion profiles for Zn(II)-Aβ40 (blue) and Ag(I)-Aβ40 (green) complexes, where the absolute chemical shift changes are plotted, obtained from a global fit analysis. The temperature dependence of the relaxation dispersion profile for two selected residues, E3 and V12, is displayed, where circles correspond to 278 (red), 281 (yellow), 284 (green), 287 (blue), and 290 K (violet) in the presence of metal ions. Black squares reflect the dynamics without any metal ions added. (c, d) The population of the NMR invisible state and the exchange rate between the two states exhibit very similar temperature dependences for Zn(II) and Ag(I). (e) The binding model is visualized where in the NMR invisible state the N-terminus encapsulates the metal ion, forming a more compact “folded” complex. Data were replotted from refs (2) and (3).

Like Cu(II), the binding site for Zn(II) in Aβ is located in the N-terminal part,^37−39^ where the first 16 residues have been assigned as the minimal binding sequence.^37^ A first study using Aβ(1–16) assigned E11 in addition to the three histidine residues as the binding ligands based on ^1^H NMR experiments.^40^ Later studies using isotope-labeled Aβ40 found no chemical shift changes or line broadening of the possible binding ligands R5, Y10, and E11, but a shift of the D1 cross-peak indicated that D1 is the fourth binding ligand.^14,38^ Yet, the Zn(II)-Aβ complex cannot be considered to be a static coordination but has been found to be highly dynamic.^2,38,39^ The dynamic nature is manifested by a signal loss of the ^1^H–^15^N HSQC NMR spectrum due to chemical exchange between a free NMR-visible state and a metal-bound NMR-invisible state (Figure 1b). First, an increase in the differences of the amide H^N^ R_1_ and R_2_ rates was reported for Aβ40,^38^ which was later confirmed by another study including a more comprehensive set of relaxation parameters as well as Aβ42.^39^ These reports demonstrated exchange dynamics on an intermediate NMR time scale. Applying ^15^N Carr–Purcell–Meiboom–Gill (CPMG) NMR relaxation dispersion experiments, the exchanging system can be quantified, revealing the exchange rate, the population of the bound state, and the chemical shift differences between the two states. Eight N-terminal residues exhibited significant amplitudes in the relaxation dispersion profiles (Figure 3a) with a temperature-dependent exchange rate of ca. 300 to 800 s^–1^ (Figure 3d).^2^ The NMR invisible state is populated only to ∼3 to 7% and decreases with increasing temperature (Figure 3c). NMR pulsed field gradient diffusion measurements indicated a more compact complex when Zn(II) is bound to Aβ compared to the metal-free state (Figure 1e), suggesting that in the NMR-invisible state the N-terminal part is folded around the Zn(II) ion.^2^ Interestingly, the rate-limiting step is presumably not the metal binding itself but the folding around the Zn(II) ion, and the population of the NMR-invisible state reflects the folded state, which encapsulates the Zn(II) ion (Figure 3e).^2^ A thermodynamic analysis revealed that the Gibbs free energy for the folding process is positive for all temperatures, meaning that the folded state is only marginally stable.^2^

### 2.3 Silver Ions Ag(I)

Similarly to Cu(I), the three histidines are involved in Ag(I) coordination as reported for the Aβ(1-16)-Ag(I) complex using ^1^H-detected solution NMR.^36^ These results were confirmed using full-length ^13^C–^15^N-labeled Aβ40 and ^1^H–^15^N and ^1^H–^13^C HSQC NMR analysis, where the D1 might be an additional binding ligand, similar to that for Zn(II).^3^ Control experiments using the triple histidine mutant H6A,H13A,H14A did not show any binding of Ag(I), stressing the importance of the histidines in Ag(I) coordination.^3^

NMR diffusion measurements exhibited a more compact metal bound state with a very similar value for the diffusion coefficient as reported for Zn(II) (Figure 1e).^2,3^ In contrast to Zn(II), significant chemical shift changes in 2D NMR ^1^H–^15^N HSQC experiments were observed in addition to NMR signal attenuation in the regions around the potential binding ligands.^3^ These residues also showed high amplitudes in ^15^N CPMG relaxation dispersion profiles (Figure 3b), where the fitted chemical shift difference between the NMR-visible and invisible states correlates with the observed ^1^H–^15^N HSQC chemical shift changes, supporting that both observations can be described by the same model.^3^ The exchange rate and population of the NMR-invisible state were found to be in the same range and show the same temperature dependence as observed for Zn(II) (Figure 3c,d).^2,3^

### 2.4 Other Investigated Metal Ions

Besides the most common physiological metal ions Cu(II) and Zn(II), physiological iron ions, Fe(II), have been reported to bind to Aβ as well as other metal ions such as Mn(II), Pb(IV), Co(II), etc., where typically the three histidines act as binding ligands. Aβ interactions with these metal ions are discussed in the literature.^13,32,41,42^

### 2.5 Common Binding Features

The in-depth-investigated metal ions Cu(II), Zn(II), and Ag(I) bind to Aβ and share several binding features (Table 2). They exhibit similar binding sites and form a more compact metal ion-bound complex with Aβ40, where the N-terminal region is wrapped around the metal ion. Remarkably, the “folded” metal-Aβ complex is not static but in dynamic exchange with the “unfolded state”.

**Table 2:** Comparison of Metal Binding Characteristics for Zn(II), Ag(I), and Cu(II) and Their Effect on Aβ Aggregation

	**Cu(II)**	**Zn(II)**	**Ag(I)**
**Metal binding**			
Binding ligands	D1 (NH_2_ terminus), D1 (CO backbone group), H6 (N_ε_), H13 or H14 (N_δ_), two modes^33,43,44^	H6, H13, H14, and D1^37−39^	H6, H13 or H14^3,36^
Chemical shift changes in ^1^H–^15^N HSQC	Residues 18–21^1^	Not observed^2,38,39^	Residues 3–21^3^
NMR diffusion	More compact state in the presence of metal ions,^1−3,34^ exhibiting the same relative diffusion coefficient^1^		
Chemical exchange process between NMR visible and invisible states	Not observable due to paramagnetic relaxation^1^	^15^N CPMG profiles for residues 3–20^2^	^15^N CPMG profiles for residues 3–16^3^
**Aggregation kinetics**			
Impact on Aβ aggregation	Retardation^1,20^	Retardation^2,45^	Retardation^3^
Predominantly affected nucleation process	Elongation rate, *k*_*+*_^1^	Elongation rate, *k*_*+*_^2^	Elongation rate, *k*_*+*_^3^
K_D,app_ [μM] to monomeric peptides from inhibition of elongation rates^1−3^	0.8 ± 0.3	1.2 ± 0.2	3.5 ± 0.4

## 3 Effect of Metal Ions on Aβ Aggregation Kinetics

### 3.1 Effect on Bulk Aggregation

Metal ions have been reported to accelerate or slow down Aβ aggregation depending on the overall metal ion concentration and metal:Aβ ratio, as discussed in several review articles.^13,16,20,46^ While Cu(II), Zn(II), and Ag(I) were shown to retard Aβ fibrillization at low metal ion concentration, at high concentrations Aβ aggregation can be promoted, resulting in the formation of amorphous aggregates.^1−3,13,16,20^ Notably, the Aβ aggregation kinetics experiments discussed here were performed at constant Aβ concentration, making the metal concentration and metal:Aβ ratio interchangeable. To quantify the effect of metal ions, an empirical description of the aggregation kinetics of Aβ can be applied, where the aggregation traces are described by a sigmoidal function. The kinetic curve is determined by the aggregation half time τ_1/2_, which reflects the time when 50% of the initial monomers are converted to fibrils, and the maximal slope of the curve, *r*_*max*_, the initial signal intensity, *F*_0_, and the final signal intensity *A*, are given by eq 1.
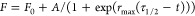
1Here, the aggregation half time τ_1/2_ has been proven to be a robust measure of the concentration-dependent inhibition effect of metal ions at low concentrations, e.g., where no amorphous aggregate formation is promoted. In addition to a prolongation of τ_1/2_, a decrease in the maximal slope of the aggregation traces has been reported.^1−3^ To elucidate details of the molecular mechanism of the inhibitory effect, a more comprehensive model needs to be introduced, including the contribution of different nucleation events, *vide infra*.

### 3.2 Morphology of Mature Fibrils in the Presence of Metal Ions

To elucidate the morphology of the final Aβ fibril (i.e., at the end of the aggregation kinetics), transmission electron microscopy (TEM) and atomic force microscopy (AFM) images can be recorded to pinpoint the potential difference of Aβ fibrils formed in the absence and presence of metal ions. At low metal ion concentrations (i.e., under conditions where a clear inhibitory effect is observable in aggregation kinetics experiments), Aβ fibril morphology appears to be similar in the presence of metal as compared to Aβ fibrils alone.^1−3^ These observations were confirmed by Fourier transform infrared (FTIR) measurements, showing similar FTIR spectra of the final aggregation products with and without Zn(II) ions.^2^ Remarkably, while for Zn(II) and Ag(I) the final ThT intensity is basically unchanged, for Cu(II) a clear decrease in signal intensity was reported.^1^ This change is seemingly caused by Cu(II) quenching of the ThT fluorescence rather than the formation of a different fibril morphology, since the ThT signal intensity could be partially recovered by the addition of EDTA, which sequesters metal ions.^1^ A solid-state NMR study on the Cu(II)-bound Aβ40 fibril found that H13 and H14 participated in Cu(II) binding, and besides D1 and H6, the carboxyl terminal of V40, E3, and E11 can also coordinate Cu(II).^47^ The hydrophobic core region of the Aβ40 fibrils was found to be unaffected by Cu(II) binding,^47^ supporting the overall unchanged fibril morphology.

### 3.3 Modulation of Specific Microscopic Nucleation Events of Aβ Self-Assembly by Metal Ions

The nucleation process of protein self-assembly can generally be described quantitatively using an analytical solution to a set of differential equations.^28,29^ This master equation includes kinetic rate constants of different microscopic nucleation events, such as primary nucleation (*k*_*n*_), secondary nucleation (*k*_*2*_), and fibril-end elongation (*k*_*+*_).^28,29^ Primary nucleation refers to the initial formation of small nucleation units from monomeric species, and fibril-end elongation represents the growth of the fibrils by the attachment of monomers to the fibril ends. During the secondary nucleation process, small nucleation units are generated on the fibril surface, which act as a catalyzer for the reaction. The analytical solution of this model gives the time dependence of the fibril mass *M*(*t*) by^28,29^
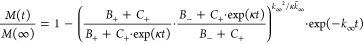
2where the global fit parameters for primary λ and secondary nucleation κ are dependent on combined nucleation rates by  and  and the additional coefficients are functions of λ and κ with *C*_±_ = ± λ^2^/2/κ^2^; ; ; and *B*_±_ = (*k*_*∞*_ ± *k̃*_*∞*_)/2/κ.

By performing a global fit analysis of a set of different aggregation kinetic measurements (i.e., including aggregation traces with varying protein and seed concentrations), the mechanism of protein self-assembly can be deciphered. For Aβ40 and Aβ42, secondary nucleation processes have been shown to govern the aggregation behavior.^30,31^

To draw conclusions from the general retardation of Aβ aggregation caused by metal ions to the microscopic mechanisms of inhibition, comprehensive sets of aggregation kinetics are required. The first experiments of this kind were performed to study the effect of Zn(II) ions on Aβ40 fibrillization.^2^ Here, the overall aggregation mechanism was unchanged in the presence of Zn(II), and the global fit analysis revealed an effect of Zn(II) on secondary nucleation and/or fibril elongation.^2^ To distinguish between these two nucleation events, highly seeded kinetic experiments can be performed. Under these conditions, primary and secondary nucleation are bypassed at the start of the reaction, which facilitates directly deducing the elongation rate from the initial slope of the aggregation kinetics profiles.^48^ From these experiments, a specific reduction of the fibril-end elongation rate could by determined for Aβ40 aggregation in the presence of substoichiometric concentrations of Zn(II).

**Figure 4:**
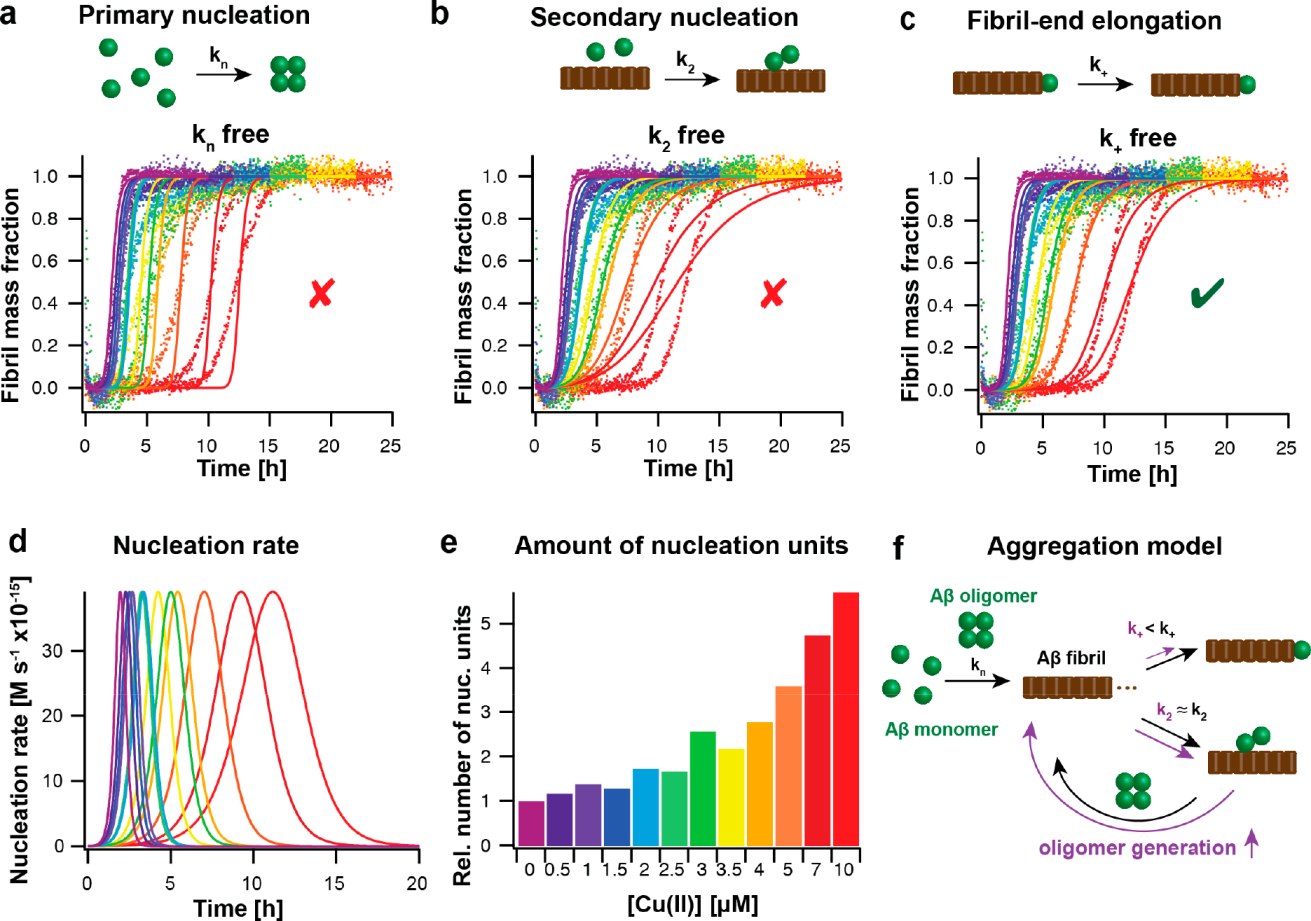
Aggregation kinetics analysis reveals a specific effect of metal ions on fibril-end elongation, leading to an enhanced oligomer generation rate. (a–c) Global fit analysis of aggregation kinetics, here shown for 3 μM Aβ42 in the presence of 0 to 10 μM Cu(II) (from violet to red colors), reveals the best fit for the elongation rate, *k*_*+*_, as the sole free fitting parameter. (d, e) From the global fit results, the nucleation rate of new nucleation units can be calculated, exhibiting a shift of the maximum of the reaction and an increased area under the curve with increasing Cu(II) concentration, corresponding to an increased number of new nucleation units. (f) Aggregation model based on kinetic analysis, showing a specific inhibitory effect on fibril-end elongation by Cu(II) (violet) that results in an increased generation rate for new nucleation units (oligomers) in the presence of Cu(II) (violet) compared to the absence of Cu(II) (black). The figure was modified with permission from ref (1). Copyright 2022 the authors. American Chemical Society.

Subsequent studies on Ag(I) and Cu(II) interactions also revealed that these metal ions have a specific inhibitory effect on fibril-end elongation obtained by combining a global fit analysis with highly seeded experiments.^1,3^ Notably, in the case of Cu(II), Aβ42 aggregation kinetics were also performed, showing the same specific inhibition as for Aβ40.^1,49^ The kinetic global fit analysis is visualized for Cu(II)-modulated Aβ42 aggregation kinetics in Figure 4.

Based on the specific effect on only one nucleation event, the fibril elongation, a model can be created describing the attachment of monomeric peptide to the fibril ends. In this model, the binding of metal ions to monomeric Aβ transiently removes monomers from the aggregation-prone pool of monomeric Aβ peptides, where the metal ion-bound Aβ peptides are no longer available for fibril-end elongation.^1,3^ This dynamic process can be quantified by an apparent dissociation constant, *K*_*D*_^*app*^. Comparing the three investigated metal ions, Cu(II) shows the strongest binding with the most prominent effect on Aβ aggregation, followed by Zn(II) and Ag(I) (Table 2). This follows the Irving–Williams series for Cu(II) and Zn(II), predicting the highest affinity for Cu(II). These findings agree well with previous studies on the dissociation constant of metal ions toward monomeric Aβ, which typically report an apparent dissociation constant, *K*_*D*_^*app*^, on the order of 10^–6^ μM (while the conditional dissociation constant is significantly lower) and the same order of the binding affinity.^13,14,19,36,38^

### 3.4 Estimations of Aβ Oligomer Generation from Aggregation Kinetics Data

An increasing line of evidence assigns the formation of oligomeric aggregation intermediates as the toxic process, not the fibrillar state as such.^4,50^ Interestingly, interactions of metal ions with oligomeric states of Aβ were reported,^51^ suggesting that metal ions interfere with the formation of Aβ oligomers, which is linked to their modulating effect on Aβ self-assembly. Indeed, the number of newly formed nucleation units can be deducted from the aggregation kinetics analysis, providing an estimate of the generation of oligomers, which convert from these small nucleation units.^52^ The nucleation rate for the formation of the nucleation units is dependent on the microscopic rate constants *k*_*n*_, *k*_*+*_, and *k*_*2*_ and is given by^52^
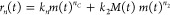
3The area under the reaction profile describes the number of newly formed nucleation units (Figure 4d,e). An inhibition of one of these rates generally results in a shift in the maximum of the reaction profile, yet the number of new nucleation units drastically differs depending on which microscopic rate is reduced.^52,53^ A modulation of *k*_*n*_ does not affect the number of nucleation units, while a specific reduction of *k*_*2*_ results in a decrease. In contrast, a specific inhibition of *k*_*+*_ causes an increase of generated Aβ oligomers.^52,53^ Hence, while the metal ions Cu(II), Zn(II), and Ag(I) prevent Aβ bulk aggregation, their specific inhibition of fibril-end elongation is related to potentially increased Aβ oligomer formation. For Cu(II), this analysis revealed a ca. 3 times increase in the relative number of newly formed Aβ oligomers at equimolar concentration of Cu(II):Aβ for both Aβ40 and Aβ42.^1^

### 3.5 Predictions of Toxic Effects from Metal Ion-Modulated Aβ Aggregation

The same approach of estimating the oligomer generation rate from kinetics data has been applied to other Aβ aggregation modulators such as molecular chaperones and antibodies, as recently reviewed in ref (53). Among them, aggregation modulators inhibiting specifically secondary nucleation, *k*_*2*_, have been of great interest since theoretical analysis of the aggregation profiles predicts a decrease in Aβ oligomer generation, potentially linked to attenuated toxic effects. Indeed, for one prominent example, the molecular chaperone-like BRICHOS domain,^54−56^ the inhibition of Aβ oligomer formation by the suppression of secondary nucleation, could be linked to reduced Aβ-associated toxicity in *in vivo* models.^53,57^ Also, a study investigating the effect of murine versions of different antibodies, which have been in clinical trials against AD, identified a specific inhibition effect on secondary nucleation, accompanied by a decreased Aβ oligomer generation rate, for the Aducanumab antibody,^10^ today approved by the FDA for AD treament.^9^

Hence, estimations of Aβ42 oligomer generation from *in vitro* aggregation kinetics seemingly correlate with modulations of toxic effects *in vivo*.^53^ While for selected molecular chaperones and antibodies this analysis has shown potential as a first prediction tool, the situation for metal ions is more complicated due to additional factors that play crucial roles. For copper, the formation of ROS, produced by Cu(II)/Cu(I) redox cycling, causes toxic products, which have been associated with neurotoxic processes in AD.^13,16^ Furthermore, metal ions are essential for a large range of different biological processes, and modulation of the metal homeostasis (e.g., by binding to accumulating amounts of Aβ aggregates) might have detrimental effects.^26^ Hence, the potential modulation of Aβ oligomer generation originated by metal ion inhibition can be considered to be a contributing factor, and more experiments are needed to evaluate the contributions of different toxic processes in detail.

**Figure 5:**
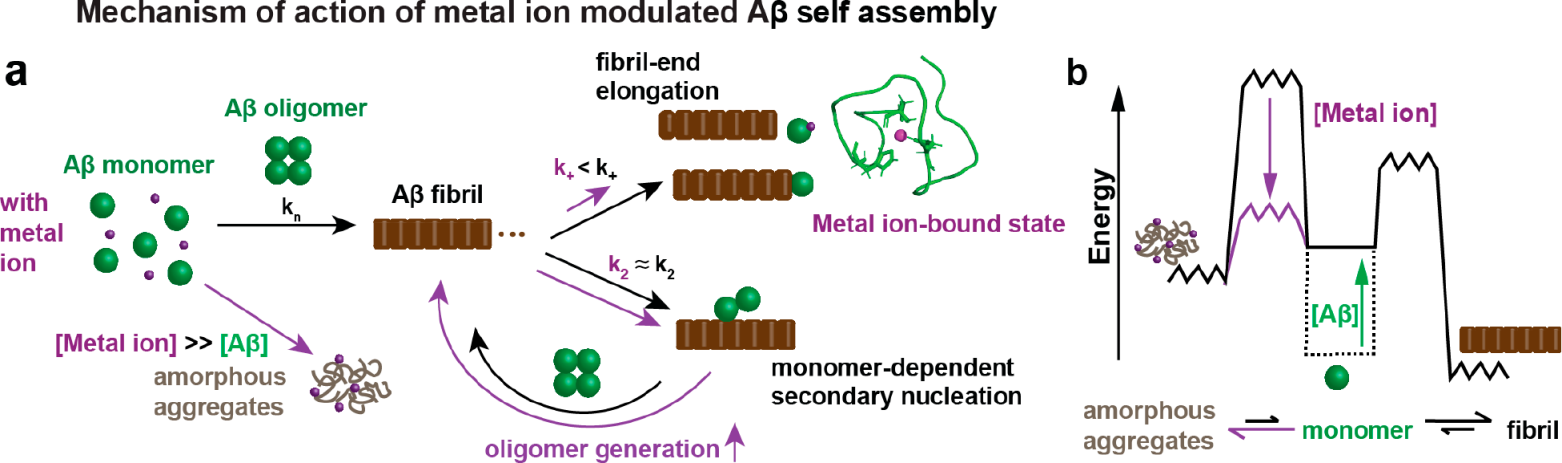
Model for mechanism of action of metal ion-modulated Aβ self-assembly. (a) Transition-metal ions, in particular referring to Cu(II), Zn(II), and Ag(I) ions, specifically prevent fibril-end elongation events by forming a seemingly aggregation-inert metal-bound Aβ complex. Inhibition of fibril elongation predicts an enhanced rate of oligomer generation. At high metal ion concentration, other aggregation processes dominate, and amorphous aggregates are formed. (b) An energy diagram shows the concentration-dependent formation of Aβ fibrils and amorphous Aβ aggregates, where an increased concentration of Aβ generally enhances aggregation and the energy barrier toward amorphous aggregate formation is determined by the metal ion concentration. The figure was modified with permission from ref (1). Copyright 2022 the authors. American Chemical Society.

## 4 Conclusions and Outlook

Transition-metal ions, here discussed for Cu(II), Zn(II), and Ag(I), interact with Aβ in a strikingly similar mechanism of action. Binding of these metal ions to monomeric Aβ causes a fold of the N-terminal part, which encapsulates the metal ion. Although the metal coordination modes might be slightly different, the three histidines are involved in binding for the investigated metal ions (Table 2). The metal-bound “folded” state is seemingly inert against aggregation, reducing the aggregation-prone pool of Aβ monomers. Due to the dynamic nature of the metal-bound state, the folding is transient and only marginally stable.^2,3^ This binding on the microscopic scale then translates to an overall retardation of bulk Aβ self-assembly on the macroscopic scale, predominately affecting the microscopic rate constant of fibril-end elongation (Table 2). While also primary and secondary nucleation are Aβ monomer-dependent, the multireaction character of the fibril elongation, consisting both of a fibril attachment and an additional “folding” step,^58^ is conceptually different from the other nucleation reactions, suggesting that the “folding” event from an unstructured monomer to a β-structured fibril element is prevented by the metal-bound state.

The microscopic insights into metal-modulated Aβ fibrillization allow a prediction of Aβ oligomer generation rates, which are presumably linked to Aβ-associated toxic effects, besides other toxic processes such as ROS formation in the case of Cu(II).^53^ The comparison to molecular chaperones exhibiting protective neurotoxic effects reveals remarkable differences with respect to the specific nucleation rate that is inhibited.^52,53,55^ Microscopic kinetic insights can hence enlarge the understanding of how metals promote potentially toxic aggregation pathways.

The here-discussed approach of combining NMR-based characterization with a detailed aggregation kinetics analysis could be transferred to other amyloid systems such as Parkinson’s-related α-synuclein or Alzheimer’s-related tau, for which specific transition-metal ion bindings were reported.^59,60^

## Abbreviations

Aβ: amyloid-β
AD: Alzheimer’s disease
CPMG: Carr–Purcell–Meiboom–Gill
CSF: cerebrospinal fluid
NMR: nuclear magnetic resonance
PRE: paramagnetic relaxation enhancement
ROS: reactive oxygen species
